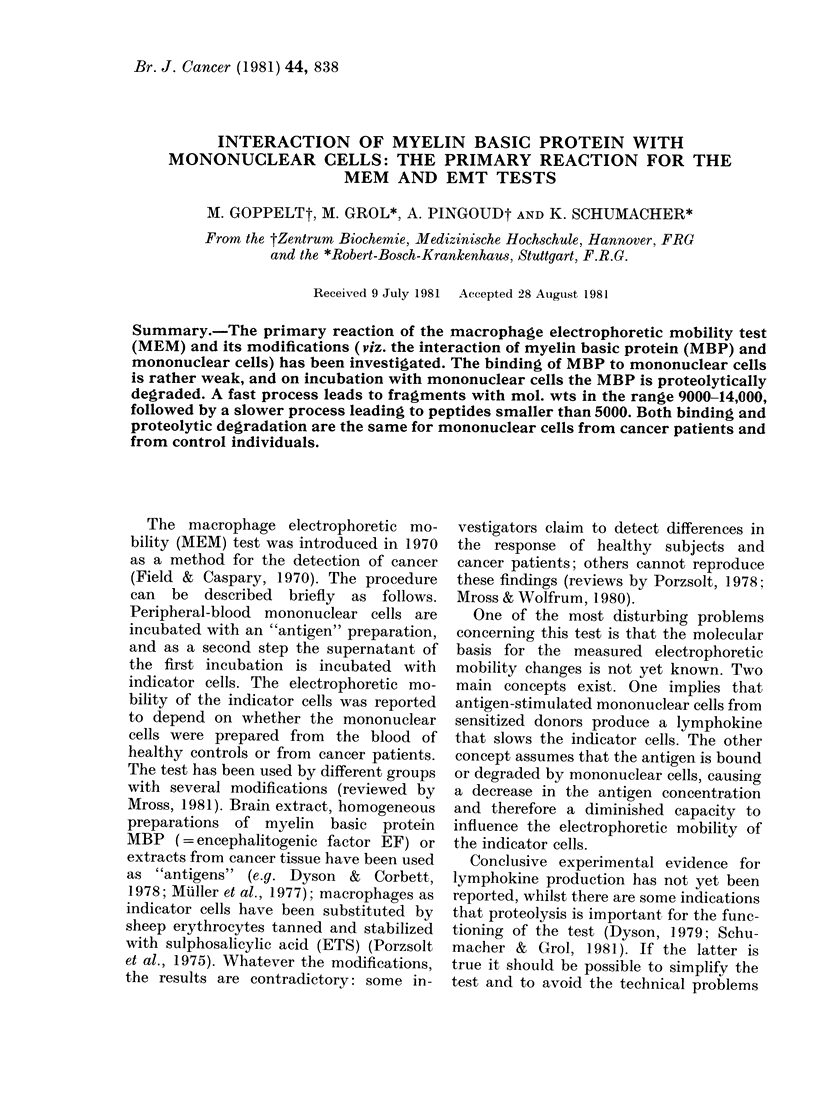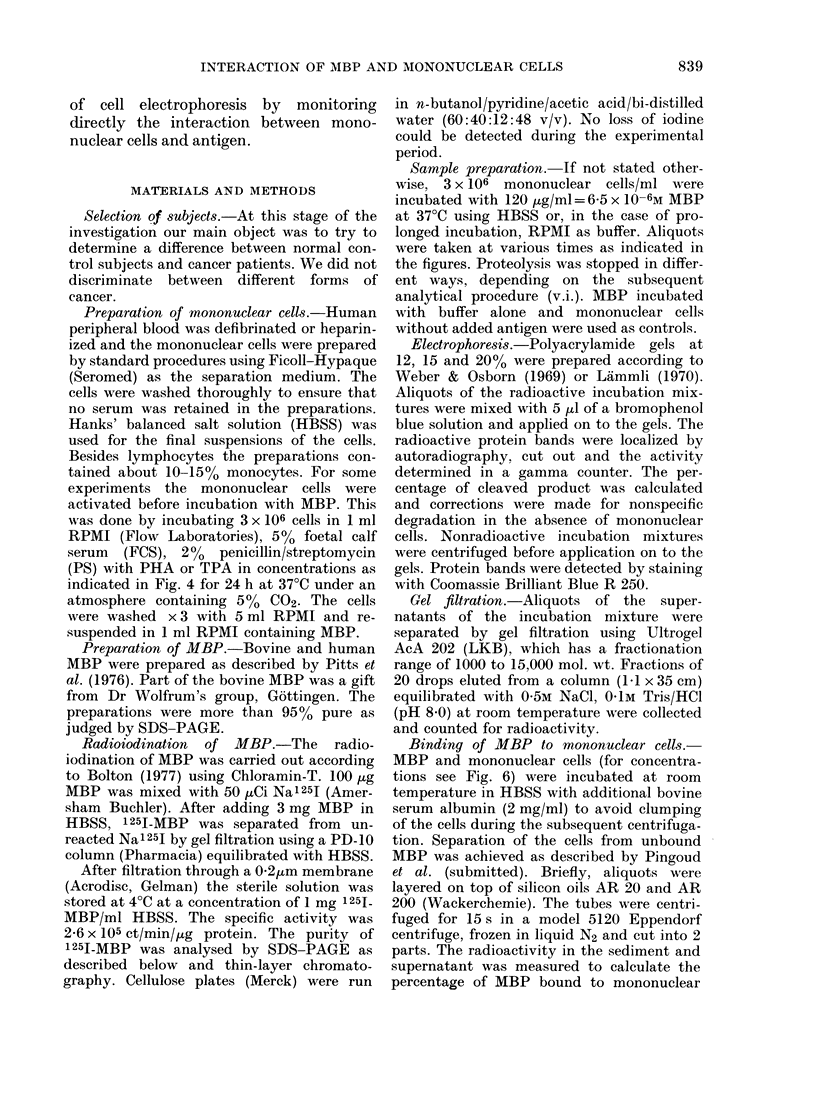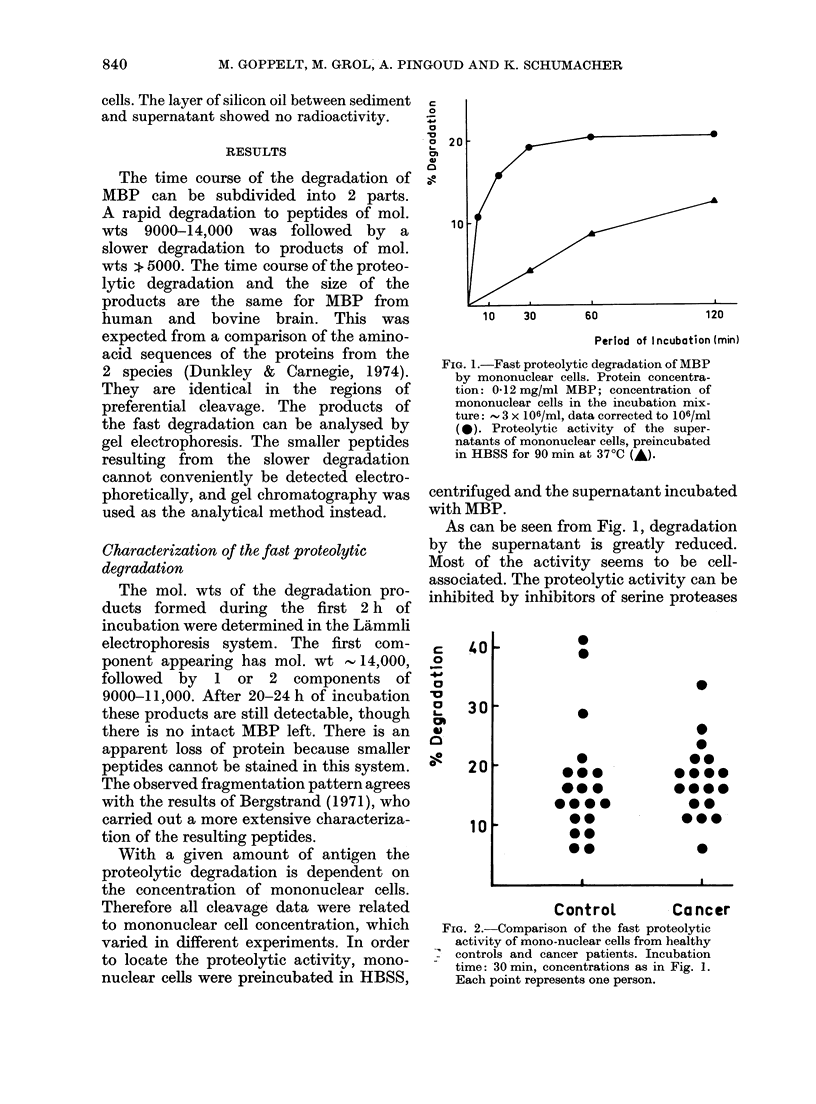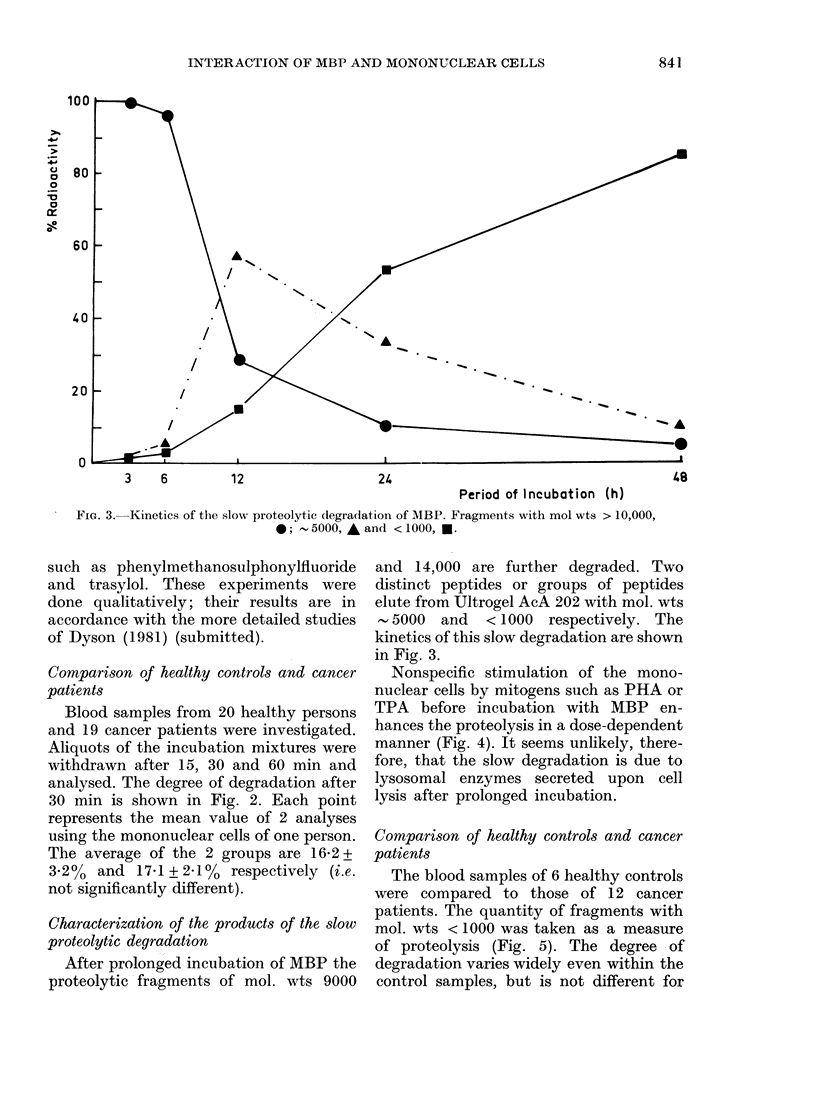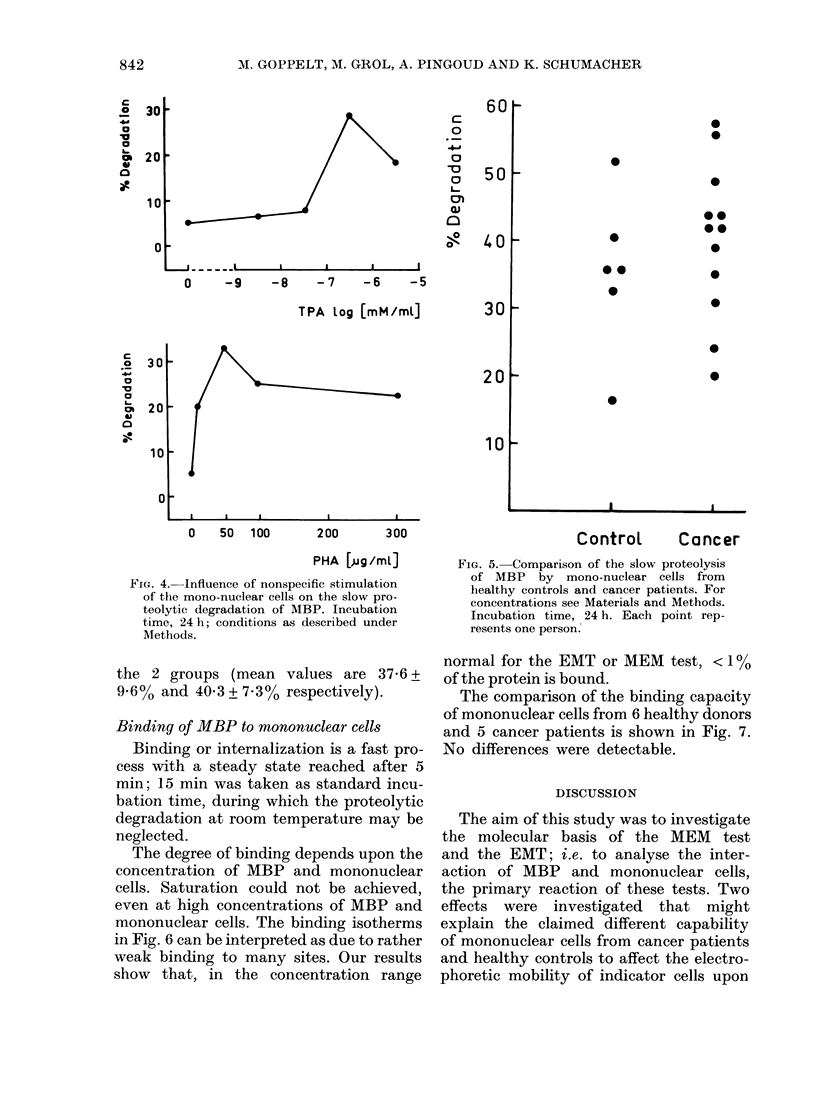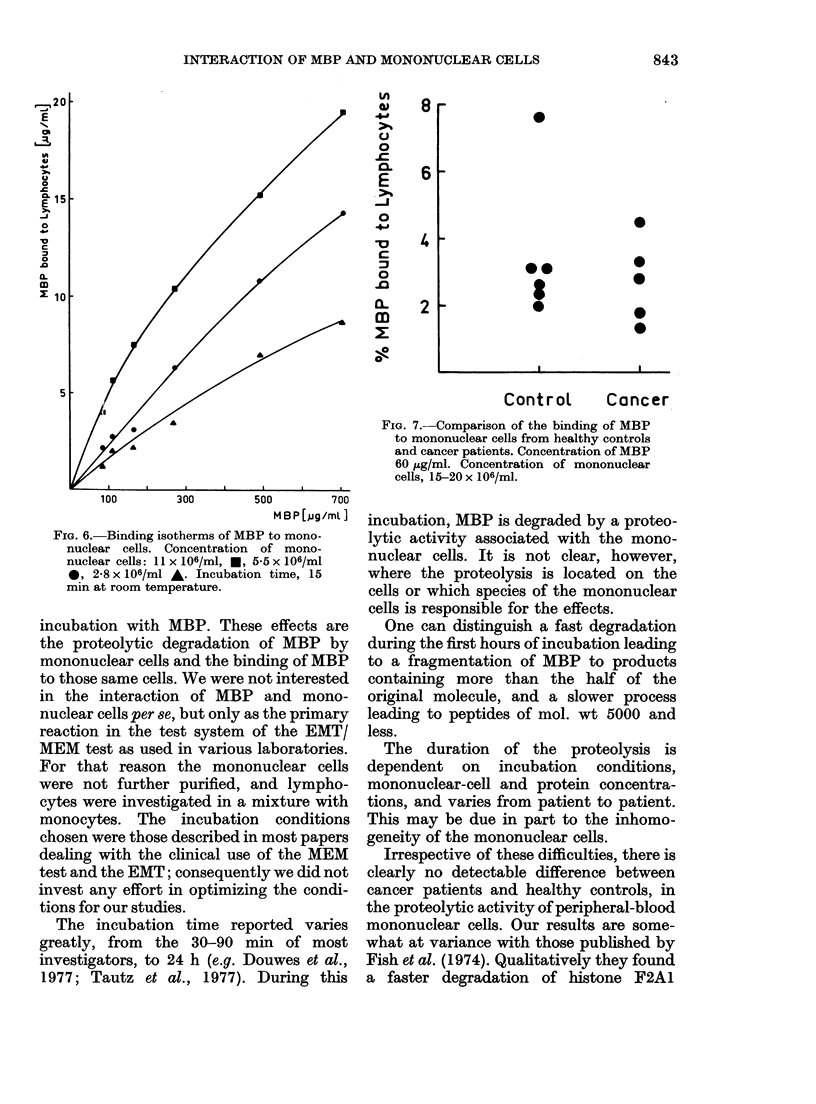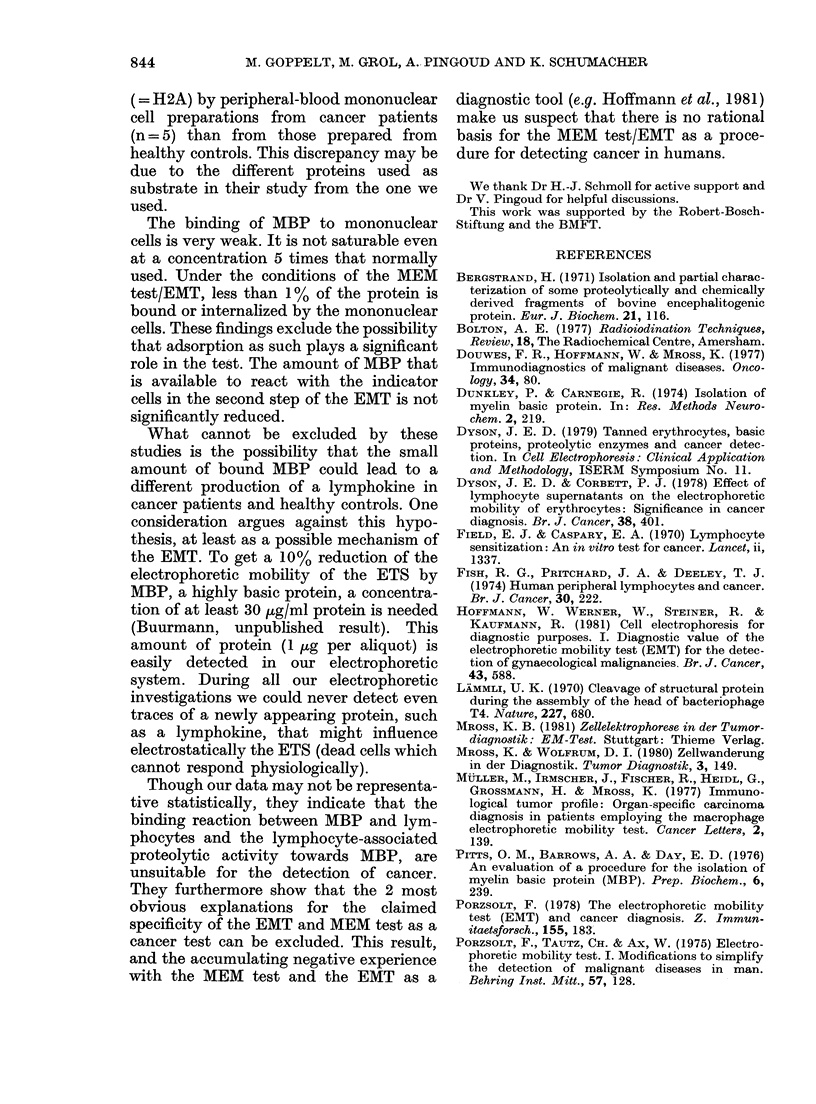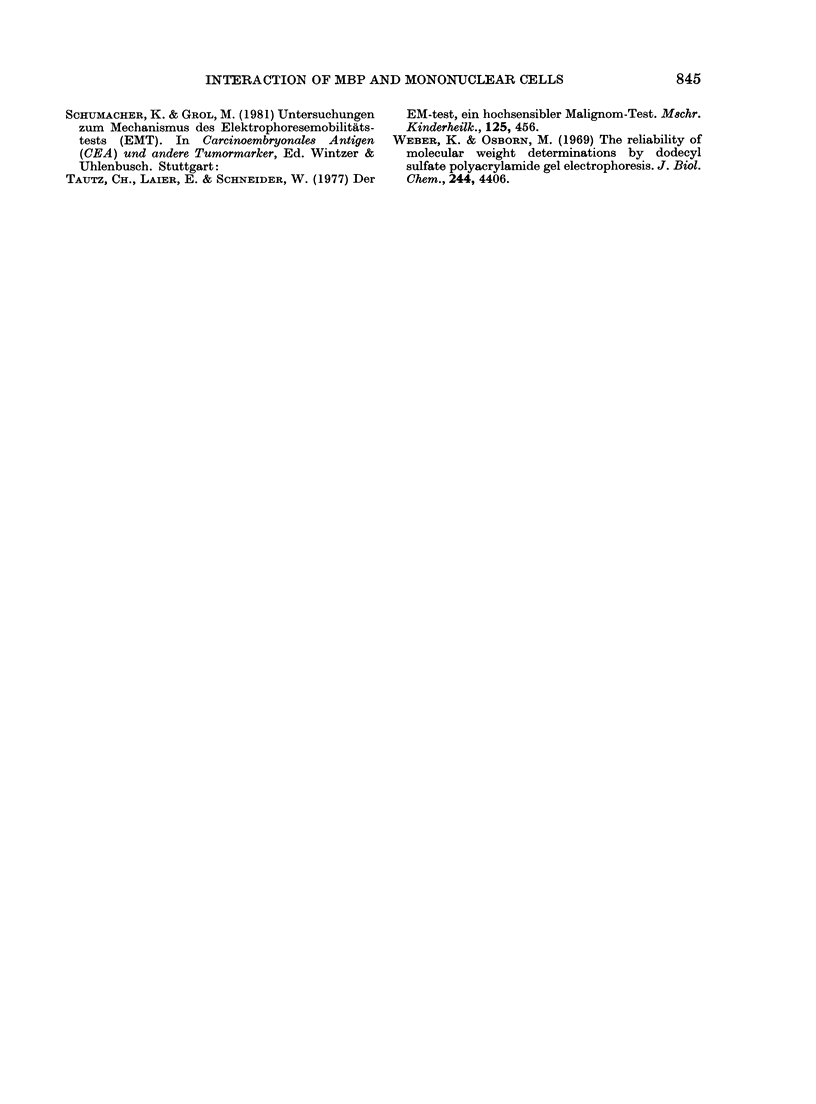# Interaction of myelin basic protein with mononuclear cells: the primary reaction for the MEM and EMT tests.

**DOI:** 10.1038/bjc.1981.282

**Published:** 1981-12

**Authors:** M. Goppelt, M. Grol, A. Pingoud, K. Schumacher

## Abstract

The primary reaction of the macrophage electrophoretic mobility test (MEM) and its modifications (viz. the interaction of myelin basic protein (MBP) and mononuclear cells) has been investigated. The binding of MBP to mononuclear cells is rather weak, and on incubation with mononuclear cells the MBP is proteolytically degraded. A fast process leads to fragments with mol. wts in the range 9000-14,000, followed by a slower process leading to peptides smaller than 5000. Both binding and proteolytic degradation are the same for mononuclear cells from cancer patients and from control individuals.


*Br. J. Cancer* (1981) **44**, 838

### INTERACTION OF MYELIN BASIC PROTEIN WITH MONONUCLEAR CELLS: THE PRIMARY REACTION FOR THE MEM AND EMT TESTS

M. GOPPELT†, M. GROL*, A. PINGOUD† AND K. SCHUMACHER*

*From the †Zentrum Biochemie, Medizinische Hochschule, Hannover, FRG and the *Robert-Bosch-Krankenhaus, Stuttgart, F.R.G.*

Received 9 July 1981 Accepted 28 August 1981

**Summary.—The primary reaction of the macrophage electrophoretic mobility test (MEM) and its modifications (*viz.* the interaction of myelin basic protein (MBP) and mononuclear cells) has been investigated. The binding of MBP to mononuclear cells is rather weak, and on incubation with mononuclear cells the MBP is proteolytically degraded. A fast process leads to fragments with mol. wts in the range 9000–14,000, followed by a slower process leading to peptides smaller than 5000. Both binding and proteolytic degradation are the same for mononuclear cells from cancer patients and from control individuals.**

The macrophage electrophoretic mobility (MEM) test was introduced in 1970 as a method for the detection of cancer (Field & Caspary, 1970). The procedure can be described briefly as follows. Peripheral-blood mononuclear cells are incubated with an "antigen" preparation, and as a second step the supernatant of the first incubation is incubated with indicator cells. The electrophoretic mobility of the indicator cells was reported to depend on whether the mononuclear cells were prepared from the blood of healthy controls or from cancer patients. The test has been used by different groups with several modifications (reviewed by Mross, 1981). Brain extract, homogeneous preparations of myelin basic protein MBP (=encephalitogenic factor EF) or extracts from cancer tissue have been used as "antigens" (*e.g.* Dyson & Corbett, 1978; Müller *et al.*, 1977); macrophages as indicator cells have been substituted by sheep erythrocytes tanned and stabilized with sulphosalicylic acid (ETS) (Porzsolt *et al.*, 1975). Whatever the modifications, the results are contradictory: some investigators claim to detect differences in the response of healthy subjects and cancer patients; others cannot reproduce these findings (reviews by Porzsolt, 1978; Mross & Wolfrum, 1980).

One of the most disturbing problems concerning this test is that the molecular basis for the measured electrophoretic mobility changes is not yet known. Two main concepts exist. One implies that antigen-stimulated mononuclear cells from sensitized donors produce a lymphokine that slows the indicator cells. The other concept assumes that the antigen is bound or degraded by mononuclear cells, causing a decrease in the antigen concentration and therefore a diminished capacity to influence the electrophoretic mobility of the indicator cells.

Conclusive experimental evidence for lymphokine production has not yet been reported, whilst there are some indications that proteolysis is important for the functioning of the test (Dyson, 1979; Schumacher & Grol, 1981). If the latter is true it should be possible to simplify the test and to avoid the technical problems of cell electrophoresis by monitoring directly the interaction between mononuclear cells and antigen.

### MATERIALS AND METHODS

*Selection of subjects.*—At this stage of the investigation our main object was to try to determine a difference between normal control subjects and cancer patients. We did not discriminate between different forms of cancer.

*Preparation of mononuclear cells.*—Human peripheral blood was defibrinated or heparinized and the mononuclear cells were prepared by standard procedures using Ficoll–Hypaque (Seromed) as the separation medium. The cells were washed thoroughly to ensure that no serum was retained in the preparations. Hanks' balanced salt solution (HBSS) was used for the final suspensions of the cells. Besides lymphocytes the preparations contained about 10–15% monocytes. For some experiments the mononuclear cells were activated before incubation with MBP. This was done by incubating $3 \times 10^6$ cells in 1 ml RPMI (Flow Laboratories), 5% foetal calf serum (FCS), 2% penicillin/streptomycin (PS) with PHA or TPA in concentrations as indicated in Fig. 4 for 24 h at 37°C under an atmosphere containing 5% $CO_2$. The cells were washed $\times 3$ with 5 ml RPMI and resuspended in 1 ml RPMI containing MBP.

*Preparation of MBP.*—Bovine and human MBP were prepared as described by Pitts *et al.* (1976). Part of the bovine MBP was a gift from Dr Wolfrum's group, Göttingen. The preparations were more than 95% pure as judged by SDS–PAGE.

*Radioiodination of MBP.*—The radioiodination of MBP was carried out according to Bolton (1977) using Chloramin-T. 100 $\mu$g MBP was mixed with 50 $\mu$Ci $Na^{125}I$ (Amersham Buchler). After adding 3 mg MBP in HBSS, $^{125}I$-MBP was separated from unreacted $Na^{125}I$ by gel filtration using a PD-10 column (Pharmacia) equilibrated with HBSS.

After filtration through a 0·2$\mu$m membrane (Acrodisc, Gelman) the sterile solution was stored at 4°C at a concentration of 1 mg $^{125}I$-MBP/ml HBSS. The specific activity was $2{\cdot}6 \times 10^5$ ct/min/$\mu$g protein. The purity of $^{125}I$-MBP was analysed by SDS–PAGE as described below and thin-layer chromatography. Cellulose plates (Merck) were run in *n*-butanol/pyridine/acetic acid/bi-distilled water (60:40:12:48 v/v). No loss of iodine could be detected during the experimental period.

*Sample preparation.*—If not stated otherwise, $3 \times 10^6$ mononuclear cells/ml were incubated with 120 $\mu$g/ml $= 6{\cdot}5 \times 10^{-6}$M MBP at 37°C using HBSS or, in the case of prolonged incubation, RPMI as buffer. Aliquots were taken at various times as indicated in the figures. Proteolysis was stopped in different ways, depending on the subsequent analytical procedure (v.i.). MBP incubated with buffer alone and mononuclear cells without added antigen were used as controls.

*Electrophoresis.*—Polyacrylamide gels at 12, 15 and 20% were prepared according to Weber & Osborn (1969) or Lämmli (1970). Aliquots of the radioactive incubation mixtures were mixed with 5 $\mu$l of a bromophenol blue solution and applied on to the gels. The radioactive protein bands were localized by autoradiography, cut out and the activity determined in a gamma counter. The percentage of cleaved product was calculated and corrections were made for nonspecific degradation in the absence of mononuclear cells. Nonradioactive incubation mixtures were centrifuged before application on to the gels. Protein bands were detected by staining with Coomassie Brilliant Blue R 250.

*Gel filtration.*—Aliquots of the supernatants of the incubation mixture were separated by gel filtration using Ultrogel AcA 202 (LKB), which has a fractionation range of 1000 to 15,000 mol. wt. Fractions of 20 drops eluted from a column ($1{\cdot}1 \times 35$ cm) equilibrated with 0·5M NaCl, 0·1M Tris/HCl (pH 8·0) at room temperature were collected and counted for radioactivity.

*Binding of MBP to mononuclear cells.*—MBP and mononuclear cells (for concentrations see Fig. 6) were incubated at room temperature in HBSS with additional bovine serum albumin (2 mg/ml) to avoid clumping of the cells during the subsequent centrifugation. Separation of the cells from unbound MBP was achieved as described by Pingoud *et al.* (submitted). Briefly, aliquots were layered on top of silicon oils AR 20 and AR 200 (Wackerchemie). The tubes were centrifuged for 15 s in a model 5120 Eppendorf centrifuge, frozen in liquid $N_2$ and cut into 2 parts. The radioactivity in the sediment and supernatant was measured to calculate the percentage of MBP bound to mononuclear cells. The layer of silicon oil between sediment and supernatant showed no radioactivity.

### RESULTS

The time course of the degradation of MBP can be subdivided into 2 parts. A rapid degradation to peptides of mol. wts 9000–14,000 was followed by a slower degradation to products of mol. wts ≯ 5000. The time course of the proteolytic degradation and the size of the products are the same for MBP from human and bovine brain. This was expected from a comparison of the amino-acid sequences of the proteins from the 2 species (Dunkley & Carnegie, 1974). They are identical in the regions of preferential cleavage. The products of the fast degradation can be analysed by gel electrophoresis. The smaller peptides resulting from the slower degradation cannot conveniently be detected electrophoretically, and gel chromatography was used as the analytical method instead.

### *Characterization of the fast proteolytic degradation*

The mol. wts of the degradation products formed during the first 2 h of incubation were determined in the Lämmli electrophoresis system. The first component appearing has mol. wt ~14,000, followed by 1 or 2 components of 9000–11,000. After 20–24 h of incubation these products are still detectable, though there is no intact MBP left. There is an apparent loss of protein because smaller peptides cannot be stained in this system. The observed fragmentation pattern agrees with the results of Bergstrand (1971), who carried out a more extensive characterization of the resulting peptides.

With a given amount of antigen the proteolytic degradation is dependent on the concentration of mononuclear cells. Therefore all cleavage data were related to mononuclear cell concentration, which varied in different experiments. In order to locate the proteolytic activity, mononuclear cells were preincubated in HBSS,

FIG. 1.—Fast proteolytic degradation of MBP by mononuclear cells. Protein concentration: 0·12 mg/ml MBP; concentration of mononuclear cells in the incubation mixture: ~3 × $10^6$/ml, data corrected to $10^6$/ml (●). Proteolytic activity of the supernatants of mononuclear cells, preincubated in HBSS for 90 min at 37°C (▲).

centrifuged and the supernatant incubated with MBP.

As can be seen from Fig. 1, degradation by the supernatant is greatly reduced. Most of the activity seems to be cell-associated. The proteolytic activity can be inhibited by inhibitors of serine proteases

FIG. 2.—Comparison of the fast proteolytic activity of mono-nuclear cells from healthy controls and cancer patients. Incubation time: 30 min, concentrations as in Fig. 1. Each point represents one person.

FIG. 3.—Kinetics of the slow proteolytic degradation of MBP. Fragments with mol wts >10,000, ●; ~5000, ▲ and <1000, ■.

such as phenylmethanosulphonylfluoride and trasylol. These experiments were done qualitatively; their results are in accordance with the more detailed studies of Dyson (1981) (submitted).

*Comparison of healthy controls and cancer patients*

Blood samples from 20 healthy persons and 19 cancer patients were investigated. Aliquots of the incubation mixtures were withdrawn after 15, 30 and 60 min and analysed. The degree of degradation after 30 min is shown in Fig. 2. Each point represents the mean value of 2 analyses using the mononuclear cells of one person. The average of the 2 groups are $16{\cdot}2 \pm 3{\cdot}2\%$ and $17{\cdot}1 \pm 2{\cdot}1\%$ respectively (*i.e.* not significantly different).

*Characterization of the products of the slow proteolytic degradation*

After prolonged incubation of MBP the proteolytic fragments of mol. wts 9000 and 14,000 are further degraded. Two distinct peptides or groups of peptides elute from Ultrogel AcA 202 with mol. wts ~5000 and <1000 respectively. The kinetics of this slow degradation are shown in Fig. 3.

Nonspecific stimulation of the mononuclear cells by mitogens such as PHA or TPA before incubation with MBP enhances the proteolysis in a dose-dependent manner (Fig. 4). It seems unlikely, therefore, that the slow degradation is due to lysosomal enzymes secreted upon cell lysis after prolonged incubation.

*Comparison of healthy controls and cancer patients*

The blood samples of 6 healthy controls were compared to those of 12 cancer patients. The quantity of fragments with mol. wts <1000 was taken as a measure of proteolysis (Fig. 5). The degree of degradation varies widely even within the control samples, but is not different for

Fig. 4.—Influence of nonspecific stimulation of the mono-nuclear cells on the slow proteolytic degradation of MBP. Incubation time, 24 h; conditions as described under Methods.

Fig. 5.—Comparison of the slow proteolysis of MBP by mono-nuclear cells from healthy controls and cancer patients. For concentrations see Materials and Methods. Incubation time, 24 h. Each point represents one person.

the 2 groups (mean values are $37{\cdot}6 \pm 9{\cdot}6\%$ and $40{\cdot}3 \pm 7{\cdot}3\%$ respectively).

*Binding of MBP to mononuclear cells*

Binding or internalization is a fast process with a steady state reached after 5 min; 15 min was taken as standard incubation time, during which the proteolytic degradation at room temperature may be neglected.

The degree of binding depends upon the concentration of MBP and mononuclear cells. Saturation could not be achieved, even at high concentrations of MBP and mononuclear cells. The binding isotherms in Fig. 6 can be interpreted as due to rather weak binding to many sites. Our results show that, in the concentration range normal for the EMT or MEM test, $< 1\%$ of the protein is bound.

The comparison of the binding capacity of mononuclear cells from 6 healthy donors and 5 cancer patients is shown in Fig. 7. No differences were detectable.

### DISCUSSION

The aim of this study was to investigate the molecular basis of the MEM test and the EMT; *i.e.* to analyse the interaction of MBP and mononuclear cells, the primary reaction of these tests. Two effects were investigated that might explain the claimed different capability of mononuclear cells from cancer patients and healthy controls to affect the electrophoretic mobility of indicator cells upon

FIG. 6.—Binding isotherms of MBP to mononuclear cells. Concentration of mononuclear cells: 11 × 10⁶/ml, ■, 5·5 × 10⁶/ml ●, 2·8 × 10⁶/ml ▲. Incubation time, 15 min at room temperature.

FIG. 7.—Comparison of the binding of MBP to mononuclear cells from healthy controls and cancer patients. Concentration of MBP 60 μg/ml. Concentration of mononuclear cells, 15–20 × 10⁶/ml.

incubation with MBP. These effects are the proteolytic degradation of MBP by mononuclear cells and the binding of MBP to those same cells. We were not interested in the interaction of MBP and mononuclear cells *per se*, but only as the primary reaction in the test system of the EMT/MEM test as used in various laboratories. For that reason the mononuclear cells were not further purified, and lymphocytes were investigated in a mixture with monocytes. The incubation conditions chosen were those described in most papers dealing with the clinical use of the MEM test and the EMT; consequently we did not invest any effort in optimizing the conditions for our studies.

The incubation time reported varies greatly, from the 30–90 min of most investigators, to 24 h (*e.g.* Douwes *et al.*, 1977; Tautz *et al.*, 1977). During this incubation, MBP is degraded by a proteolytic activity associated with the mononuclear cells. It is not clear, however, where the proteolysis is located on the cells or which species of the mononuclear cells is responsible for the effects.

One can distinguish a fast degradation during the first hours of incubation leading to a fragmentation of MBP to products containing more than the half of the original molecule, and a slower process leading to peptides of mol. wt 5000 and less.

The duration of the proteolysis is dependent on incubation conditions, mononuclear-cell and protein concentrations, and varies from patient to patient. This may be due in part to the inhomogeneity of the mononuclear cells.

Irrespective of these difficulties, there is clearly no detectable difference between cancer patients and healthy controls, in the proteolytic activity of peripheral-blood mononuclear cells. Our results are somewhat at variance with those published by Fish *et al.* (1974). Qualitatively they found a faster degradation of histone F2A1

(= H2A) by peripheral-blood mononuclear cell preparations from cancer patients (n = 5) than from those prepared from healthy controls. This discrepancy may be due to the different proteins used as substrate in their study from the one we used.

The binding of MBP to mononuclear cells is very weak. It is not saturable even at a concentration 5 times that normally used. Under the conditions of the MEM test/EMT, less than 1% of the protein is bound or internalized by the mononuclear cells. These findings exclude the possibility that adsorption as such plays a significant role in the test. The amount of MBP that is available to react with the indicator cells in the second step of the EMT is not significantly reduced.

What cannot be excluded by these studies is the possibility that the small amount of bound MBP could lead to a different production of a lymphokine in cancer patients and healthy controls. One consideration argues against this hypothesis, at least as a possible mechanism of the EMT. To get a 10% reduction of the electrophoretic mobility of the ETS by MBP, a highly basic protein, a concentration of at least 30 $\mu$g/ml protein is needed (Buurmann, unpublished result). This amount of protein (1 $\mu$g per aliquot) is easily detected in our electrophoretic system. During all our electrophoretic investigations we could never detect even traces of a newly appearing protein, such as a lymphokine, that might influence electrostatically the ETS (dead cells which cannot respond physiologically).

Though our data may not be representative statistically, they indicate that the binding reaction between MBP and lymphocytes and the lymphocyte-associated proteolytic activity towards MBP, are unsuitable for the detection of cancer. They furthermore show that the 2 most obvious explanations for the claimed specificity of the EMT and MEM test as a cancer test can be excluded. This result, and the accumulating negative experience with the MEM test and the EMT as a diagnostic tool (*e.g.* Hoffmann *et al.*, 1981) make us suspect that there is no rational basis for the MEM test/EMT as a procedure for detecting cancer in humans.

We thank Dr H.-J. Schmoll for active support and Dr V. Pingoud for helpful discussions.

This work was supported by the Robert-Bosch-Stiftung and the BMFT.

### REFERENCES

BERGSTRAND, H. (1971) Isolation and partial characterization of some proteolytically and chemically derived fragments of bovine encephalitogenic protein. *Eur. J. Biochem.* **21**, 116.

BOLTON, A. E. (1977) *Radioiodination Techniques, Review*, **18**, The Radiochemical Centre, Amersham.

DOUWES, F. R., HOFFMANN, W. & MROSS, K. (1977) Immunodiagnostics of malignant diseases. *Oncology*, **34**, 80.

DUNKLEY, P. & CARNEGIE, R. (1974) Isolation of myelin basic protein. In: *Res. Methods Neurochem.* **2**, 219.

DYSON, J. E. D. (1979) Tanned erythrocytes, basic proteins, proteolytic enzymes and cancer detection. In *Cell Electrophoresis: Clinical Application and Methodology*, ISERM Symposium No. 11.

DYSON, J. E. D. & CORBETT, P. J. (1978) Effect of lymphocyte supernatants on the electrophoretic mobility of erythrocytes: Significance in cancer diagnosis. *Br. J. Cancer*, **38**, 401.

FIELD, E. J. & CASPARY, E. A. (1970) Lymphocyte sensitization: An *in vitro* test for cancer. *Lancet*, ii, 1337.

FISH, R. G., PRITCHARD, J. A. & DEELEY, T. J. (1974) Human peripheral lymphocytes and cancer. *Br. J. Cancer*, **30**, 222.

HOFFMANN, W. WERNER, W., STEINER, R. & KAUFMANN, R. (1981) Cell electrophoresis for diagnostic purposes. I. Diagnostic value of the electrophoretic mobility test (EMT) for the detection of gynaecological malignancies. *Br. J. Cancer*, **43**, 588.

LÄMMLI, U. K. (1970) Cleavage of structural protein during the assembly of the head of bacteriophage T4. *Nature*, **227**, 680.

MROSS, K. B. (1981) *Zellelektrophorese in der Tumordiagnostik: EM-Test.* Stuttgart: Thieme Verlag.

MROSS, K. & WOLFRUM, D. I. (1980) Zellwanderung in der Diagnostik. *Tumor Diagnostik*, **3**, 149.

MÜLLER, M., IRMSCHER, J., FISCHER, R., HEIDL, G., GROSSMANN, H. & MROSS, K. (1977) Immunological tumor profile: Organ-specific carcinoma diagnosis in patients employing the macrophage electrophoretic mobility test. *Cancer Letters*, **2**, 139.

PITTS, O. M., BARROWS, A. A. & DAY, E. D. (1976) An evaluation of a procedure for the isolation of myelin basic protein (MBP). *Prep. Biochem.*, **6**, 239.

PORZSOLT, F. (1978) The electrophoretic mobility test (EMT) and cancer diagnosis. *Z. Immunitaetsforsch.*, **155**, 183.

PORZSOLT, F., TAUTZ, CH. & AX, W. (1975) Electrophoretic mobility test. I. Modifications to simplify the detection of malignant diseases in man. *Behring Inst. Mitt.*, **57**, 128.

Schumacher, K. & Grol, M. (1981) Untersuchungen zum Mechanismus des Elektrophoresemobilitätstests (EMT). In *Carcinoembryonales Antigen (CEA) und andere Tumormarker*, Ed. Wintzer & Uhlenbusch. Stuttgart:

Tautz, Ch., Laier, E. & Schneider, W. (1977) Der EM-test, ein hochsensibler Malignom-Test. *Mschr. Kinderheilk.*, **125**, 456.

Weber, K. & Osborn, M. (1969) The reliability of molecular weight determinations by dodecyl sulfate polyacrylamide gel electrophoresis. *J. Biol. Chem.*, **244**, 4406.